# Planning for globally coordinated cessation of bivalent oral poliovirus vaccine: risks of non-synchronous cessation and unauthorized oral poliovirus vaccine use

**DOI:** 10.1186/s12879-018-3074-0

**Published:** 2018-04-10

**Authors:** Radboud J. Duintjer Tebbens, Lee M. Hampton, Kimberly M. Thompson

**Affiliations:** 1 Kid Risk, Inc., 605 N High St, #253, Columbus, OH 43215 USA; 2 Global Immunization Division, Center for Global Health, Centers for Disease Control and Prevention, Atlanta, GA USA

**Keywords:** Polio, Eradication, Risk management, Oral poliovirus vaccine, Dynamic modeling, Vaccine-derived poliovirus

## Abstract

**Background:**

Oral polio vaccine (OPV) containing attenuated serotype 2 polioviruses was globally withdrawn in 2016, and bivalent OPV (bOPV) containing attenuated serotype 1 and 3 polioviruses needs to be withdrawn after the certification of eradication of all wild polioviruses to eliminate future risks from vaccine-derived polioviruses (VDPVs). To minimize risks from VDPVs, the planning and implementation of bOPV withdrawal should build on the experience with withdrawing OPV containing serotype 2 polioviruses while taking into account similarities and differences between the three poliovirus serotypes.

**Methods:**

We explored the risks from (i) a failure to synchronize OPV cessation and (ii) unauthorized post-cessation OPV use for serotypes 1 and 3 in the context of globally-coordinated future bOPV cessation and compared the results to similar analyses for serotype 2 OPV cessation.

**Results:**

While the risks associated with a failure to synchronize cessation and unauthorized post-cessation OPV use appear to be substantially lower for serotype 3 polioviruses than for serotype 2 polioviruses, the risks for serotype 1 appear similar to those for serotype 2. Increasing population immunity to serotype 1 and 3 poliovirus transmission using pre-cessation bOPV supplemental immunization activities and inactivated poliovirus vaccine in routine immunization reduces the risks of circulating VDPVs associated with non-synchronized cessation or unauthorized OPV use.

**Conclusions:**

The Global Polio Eradication Initiative should synchronize global bOPV cessation during a similar window of time as occurred for the global cessation of OPV containing serotype 2 polioviruses and should rigorously verify the absence of bOPV in immunization systems after its cessation.

## Background

Cessation of the use of oral poliovirus vaccine (OPV) represents an essential part of the global polio eradication endgame strategy [1, 2] because OPV use can, in very rare cases, lead to the development of vaccine-derived polioviruses (VDPVs). These VDPVs can cause paralytic poliomyelitis (polio) outbreaks similar to those caused by wild polioviruses (WPVs) [3]. Following certification of the eradication of WPVs, the risks of regular continued use of OPV outweigh the benefits [4, 5]. Therefore, following the certification of the eradication of serotype 2 WPV [6], the Global Polio Eradication Initiative (GPEI) coordinated the global cessation of all use of trivalent OPV (tOPV), which contained live, attenuated serotypes 1, 2, and 3 polioviruses, and replaced it with bivalent OPV (bOPV), which contains only serotypes 1 and 3 [7]. By May 2016, all 155 countries using tOPV in 2015 had ceased use of tOPV. Although some evidence suggests limited use of tOPV after the global tOPV-bOPV switch [8], countries successfully withdrew the vast majority of tOPV [9].

If the world can successfully complete serotype 1 WPV eradication, certify the world free of WPV serotypes 1 and 3, and manage the risks associated with the cessation of use of OPV containing serotype 2 polioviruses, then it will need to proceed to bOPV cessation to stop the use of all OPV containing serotype 1 and serotype 3. As of the end of 2017, no reported cases associated with serotype 3 WPV have occurred since 2012 [10] and indigenous transmission of serotype 1 WPV persists in only three countries, i.e., Afghanistan, Nigeria, and Pakistan [11], so the eradication of WPV in the near future remains a distinct possibility.

Although eliminating future polio cases caused by VDPVs requires the cessation of OPV use [4], OPV cessation carries its own risks because population immunity to poliovirus transmission (i.e., the collective immunological protection of all individuals in a population from participation in poliovirus transmission, independent of whether they contract polio disease) starts to decline once the use of OPV ceases [12]. Low population immunity to poliovirus transmission facilitates spread of the virus, so if WPVs or VDPVs are introduced into a population with low immunity, these viruses could rapidly spread and potentially cause polio outbreaks. Such introductions could occur through release of poliovirus from a vaccine production site or laboratory or through excretion of poliovirus by individuals with certain B-cell related primary immunodeficiencies with a prolonged or chronic poliovirus infection [13]. Live, attenuated poliovirus introduced into a population with low immunity to poliovirus transmission could spread and over time evolve into circulating VDPVs (cVDPVs), which in turn could cause polio outbreaks. Such introductions could occur if a country continues general use of OPV serotypes that other countries have stopped using or if OPV is used without authorization in an area well after cessation of its general use. The GPEI synchronized the global cessation of tOPV use across and within countries. The process included an extensive monitoring effort to identify and destroy remaining tOPV to prevent introductions of live, attenuated serotype 2 polioviruses [7, 9, 14–16].

Using inactivated polio vaccine (IPV) provides another option for addressing the risks associated with cessation of OPV, but it comes with limitations. While IPV provides very effective protection from paralytic polio, it remains much less effective than OPV in preventing poliovirus transmission. Individuals vaccinated with IPV without prior infection by a live poliovirus (LPV) can be infected and participate in fecal-oral poliovirus transmission to a similar degree as completely unvaccinated individuals [17–19]. In addition, IPV costs more than OPV, and the current IPV supply is insufficient to meet the rapidly increased global demand. The recent global shortage of IPV has prevented many countries from introducing IPV and caused others to run out of their IPV stock [7, 20].

The degree to which serotypes 1 and 3 OPV viruses behave like serotype 2 OPV viruses, especially regarding their ability to spread and evolve into VDPVs, is a key consideration for the planning and implementation of bOPV cessation. Similar behavior between serotype 2 and serotypes 1 and 3 would suggest a need for similar synchronization and monitoring while a serotype with substantially lower ability to spread and cause VDPVs could be withdrawn with much looser synchronization and monitoring without substantially increasing the risks associated with the withdrawal [21]. We previously used existing models [4, 22] to explore the risks associated with a failure to globally synchronize the tOPV-bOPV switch or to fully withdraw tOPV, leading to its inadvertent use [15, 16]. We also investigated how different levels of maintenance of supplemental immunization activities (SIAs) using bOPV or intensification of bOPV SIAs prior to bOPV cessation affect the risk of creating indigenous cVDPVs before or after bOPV cessation [23]. However, the attenuated virus serotypes in OPV differ in their ability to transmit and evolve to cVDPVs. The serotypes also differ with respect to the transmissibility and neurovirulence of their cVDPVs and WPVs and with respect to the level of population immunity to transmission induced by tOPV or bOPV [24]. These differences motivate analyses to compare the OPV cessation behavior among the three serotypes. Our current study explores the risks of a failure to synchronize OPV cessation or of unauthorized post-cessation use of OPV for serotypes 1 and 3 in the context of globally coordinated future bOPV cessation. We explicitly consider the impact of variable intensities of bOPV use until bOPV cessation and the presence or absence of IPV use.

## Methods

We used previously developed models and analytical frameworks to estimate the implementation risks of OPV cessation [15, 16] and the effects of different pre-cessation vaccination strategies [23]. Specifically, we used a differential equation-based model, i.e., the DEB model [22, 25], to simulate poliovirus transmission and evolution in any given population. We further used an integrated global model for long-term poliovirus risk management, i.e., the global model [4], to characterize the global variability in poliovirus transmission and vaccination. The DEB model characterizes evolution of the live, attenuated polioviruses in OPV to VDPVs as a 20-stage reversion process. That reversion process starts with the attenuated poliovirus parent strain in OPV (stage 0) with low transmissibility (expressed as the basic reproduction number, or R_0_) and low neurovirulence (expressed as the paralysis-to-infection ratio). Through reversion, these viruses gradually evolve to fully-reverted VDPVs with assumed R_0_ and paralysis-to-infection ratio values equal to those for the homotypic WPVs. Consistent with the available evidence, the model assumed serotype-specific differences for the absolute R_0_ values of VDPVs, for the ratios of the R_0_ values of the attenuated parent strains in OPV to the R_0_ values of the homotypic WPVs (and VDPVs), and for the reversion times. For a given population, the model calculated each of these R_0_ values relative to the assumed R_0_ value for serotype 1 WPV in that population (see example in Table 1) [22, 23, 25, 26].

**Table 1:** Illustration of serotype specific properties implied by assumed model inputs [22, 49] in a population with an R_0_ of 10 for serotype 1 WPV

Property	Virus (reversion stage in the model)	Serotype		
		1	2	3
Basic reproduction number (R_0_)	OPV parent strain (stage 0)	3.7	5.0	1.9
	Partially-reverted OPV-related virus (stage 10)	7.0	7.1	4.8
	Fully-reverted VDPV or WPV (stage 19)	10	9.0	7.5
Average time (days) to reach reversion stage	OPV parent strain (stage 0)	0	0	0
	Partially-reverted OPV-related virus (stage 10)	327	215	327
	Fully-reverted VDPV or WPV (stage 19)	620.5	408	620.5

*WPV* wild poliovirus, *OPV* oral poliovirus vaccine, *VDPV* vaccine-derived poliovirus

We conducted two analyses (I and II) related to the implication of a failure to globally synchronize OPV cessation and two analyses (III and IV) related to unauthorized post-cessation use of OPV. Analyses I and II considered the vulnerability of the various subpopulations to OPV-related viruses as a function of time after the tOPV-bOPV switch (for serotype 2) or after bOPV cessation (for serotypes 1 and 3). These analyses focus on vulnerability and do not consider the likelihood of exposure to OPV-related viruses that would occur in the event of a non-synchronous cessation, which could include exposure to OPV-related viruses in different stages of reversion. In the context of high population immunity to transmission and continued OPV use, the majority of OPV-related viruses reside in very low stages of reversion because insufficient susceptible individuals exist for the sustained transmission of OPV-related viruses needed for them to evolve to higher reversion stages. With lower population immunity to transmission, OPV-related viruses in higher reversion stages may exist in relatively greater numbers. Thus, if countries do not synchronize OPV cessation, then countries that stop OPV use earlier could import OPV-related viruses in any reversion stage, depending both on chance and the population immunity to transmission in the countries that still use OPV. To convey the range of vulnerabilities to different OPV-related viruses, we show the vulnerability to reversion stages 0 (OPV parent strain), 10 (partially-reverted OPV-related virus), and/or 19 (fully-reverted VDPV). Analyses I and II used the integrated global model, which divides the world into 710 subpopulations, and focused on the 520 subpopulations that used OPV in 2013 [4]. We characterized the vulnerability of a population to the spread of polioviruses using the mixing-adjusted net reproduction number (R_n_), defined as the average number of secondary infections generated by a single infection. R_n_ accounts for the R_0_ of the poliovirus and the level of population immunity to transmission. An R_n_ of greater than 1 means that the corresponding poliovirus can sustain circulation. R_n_ values in a given population vary for different polioviruses, i.e., serotypes and reversion stages, because the corresponding R_0_ values differ, and they change over time due to seasonality in poliovirus transmissibility and changing population immunity to transmission. Unless an outbreak occurs in a population, R_n_ values continually increase following bOPV cessation due to decreasing population immunity to transmission in the absence of exposure to LPVs. The global model base case, which we adopted for analyses I and II, assumed IPV introduction in all countries by January 1, 2015, tOPV cessation on April 1, 2016, and bOPV cessation on April 1, 2019 [4]. The model assumes that any outbreaks of polio cases after OPV cessation result in an aggressive outbreak response with homotypic monovalent OPV from a stockpile [4]. We explored different outbreak response options and stockpile considerations elsewhere [27, 28].

For analysis I, we compared the vulnerability after OPV cessation among the three serotypes, assuming tOPV intensification prior to the tOPV-bOPV switch and continued high maintenance of population immunity with bOPV SIAs until bOPV cessation (Table 2, base case scenario). For analysis II, we assessed the vulnerability of different subpopulations to the spread of serotype 1 and 3 polioviruses under various strategies of bOPV use between the tOPV-bOPV switch and bOPV cessation [29]. As shown in Table 2, the medium population immunity maintenance scenario assumes two fewer annual bOPV SIAs than the high population immunity maintenance base case scenario in all subpopulations with routine immunization (RI) coverage of less than 90% and no bOPV SIAs in all subpopulations with very high RI coverage. The low population immunity maintenance scenario limits bOPV SIAs to subpopulations with ≤60% RI coverage with three non-birth bOPV doses, and for the populations still performing SIAs, further reduces the annual number of bOPV SIAs by one or two rounds compared to the medium maintenance scenario. A previous study showed that the first two scenarios suffice to prevent the emergence of indigenous serotype 1 or 3 cVDPVs before or after bOPV, but that the low population immunity maintenance scenario results in a serotype 1 cVDPV outbreak in a high-risk subpopulation [29].

**Table 2:** Annual bOPV SIA frequencies between the tOPV-bOPV switch and bOPV cessation assumed for the different population immunity maintenance scenarios, adopted from prior work [29] for the analysis of the vulnerability of populations in the event of non-synchronous bOPV cessation

Subpopulation characteristics		Annual number of bOPV SIAs for each population immunity maintenance scenario		
Routine immunization coverage with 3 or more non-birth bOPV doses	Basic reproduction number (for serotype 1 wild poliovirus)	Base case (continued high population immunity maintenance)	Medium population immunity maintenance	Low population immunity maintenance^a^
5% or 10%	Any	6	4	3
30%	Any	5	3	2
60%	10 or less	3	1	1
60%	More than 10	5	3	1
90%	Any	1	1	0
98%	10 or less	0	0	0
98%	More than 10	1	0	0

^a^ Previous research indicates that this scenario results in a circulating vaccine-derived poliovirus outbreak after bOPV cessation [29].

*bOPV* bivalent oral poliovirus vaccine, *tOPV* trivalent oral poliovirus vaccine, *SIA* supplemental immunization activity

For analyses III and IV, we use the DEB model to simulate different levels of continued OPV use after cessation in specific high-risk subpopulations from the global model [16]. In our prior analysis that focused on serotype 2 and the tOPV-bOPV switch [16], we referred to such continued use as “inadvertent use.” However, in the context of cessation of use of all OPV this inadvertent use seems less likely given the change in administration method, e.g., from oral OPV to injectable IPV. Therefore, we use the term “unauthorized use” to cover all potential contexts of continued OPV use after its cessation outside of explicit authorization to use OPV from the stockpile to respond to an outbreak.

For both analyses III and IV, we determined the minimum duration after OPV cessation of unauthorized OPV use in RI before that unauthorized use resulted in the creation of an indigenous cVDPV outbreak. Given our focus on occurrence of a cVDPV outbreak, we did not include any subsequent outbreak response. We updated the time of bOPV cessation to reflect the current expectation of bOPV cessation, specifically to the end of April, 2021. In light of the experience with tOPV withdrawal [8], we focused on unauthorized use in RI. Specifically, we modeled a constant level of unauthorized OPV use in RI over time, i.e., a rectangular scenario [16]. For serotype 2, we previously also explored an exponential decay scenario in which the level of unauthorized use declines over time [16]. Both scenarios yielded the same qualitative insight that later and more unauthorized OPV use (up to the point when that OPV use starts to become so large that it maintains higher population immunity) increases the risk of a cVDPV outbreak. Therefore, for the purpose of comparison among the serotypes we focus on the conceptually simplest rectangular scenario [16]. Recognizing that the relative differences among serotypes and the absolute risks from cVDPVs may vary among settings, but assuming that the qualitative insights remain similar for all high-risk populations, we selected two subpopulations from the broader set that we considered in analyses I and II [16]. The first high-risk subpopulation assumes properties similar to those of the general population of northern India, with an R_0_ of 13 for serotype 1 WPV, 60% RI coverage with three non-birth OPV doses, and high-quality OPV SIAs with a true coverage of 95% The second high-risk subpopulation assumes properties similar to the general population of northern Nigeria, with an R_0_ of 8 for serotype 1 WPV, 30% RI coverage with three non-birth OPV doses, and low-quality OPV SIAs with a true coverage of 50%. For both of these analyses, we assumed that the birth dose coverage equals half of the coverage with three non-birth OPV doses, that 20% of children who do not receive three or more non-birth RI OPV doses receive one non-birth dose, and that another 20% receive two non-birth doses. We optimistically assumed that children who receive one or two non-birth doses visit health services and receive IPV at the scheduled time of the third non-birth dose, which we modeled to occur at 3 months of age [30]. For the assumed RI coverage levels, this implies coverage with a single IPV dose of 76% in northern India and 58% in northern Nigeria.

Analysis III compared the implications of unauthorized post-cessation OPV use for all three serotypes. This analysis assumed three annual tOPV SIAs before the tOPV-bOPV switch and analogously three annual bOPV SIAs before bOPV cessation. The analysis also included a single dose of IPV introduced in 2015. To explore the impact of the amount of unauthorized OPV use, we considered different proportions of covered children who receive a full schedule of RI doses, i.e., birth dose and three non-birth doses, of the stopped OPV vaccine instead of the poliovirus vaccine that remains in the RI schedule, i.e., bOPV and IPV after the tOPV-bOPV switch and IPV after bOPV cessation.

Analysis IV explored the serotype 1 risks of unauthorized bOPV use under various assumptions about IPV and bOPV use until bOPV cessation. We took the same approach as for analysis III, except that we assumed that unauthorized bOPV use would only occur at the time of a post-bOPV cessation scheduled IPV dose delivered at the age of 3 months instead of at all scheduled RI visits. Thus, for analysis IV, the unauthorized use proportion represents the fraction of children in a given population covered by RI who receive a single dose of the stopped bOPV vaccine instead of a scheduled IPV dose. The base case for analysis IV assumed reduced bOPV SIA frequency of one annual SIA between tOPV cessation and the year before bOPV cessation and two bOPV SIAs in the year of bOPV cessation, i.e., 2021, as well as a single dose of IPV given in RI at 3 months. We then compared this base case to a scenario of three annual bOPV SIAs prior to bOPV cessation (labeled “High maintenance”), a hypothetical scenario of no IPV use since 2015 (labeled “No IPV from 2015”), and a scenario reflecting a decision to switch from the one IPV dose introduced in 2015 to two IPV doses at the time of bOPV cessation (labeled “Two IPV from bOPV cessation”), which assumes sufficient IPV supplies available to do so by 2021. We assumed an average per-dose take rate of IPV of 63%, i.e., corresponding to a cumulative take rate of 95% after three doses [4], and modeled the cumulative effect of any IPV doses as occurring by the time children reach the age of 3 months [30]. For the scenario “Two IPV doses from bOPV cessation”, we assumed that any child who receives at least two non-birth RI doses receives both IPV doses. The DEB model assumed full protection from paralytic polio after one or more successful IPV doses, i.e., IPV doses that stimulate an immune response. The model assumed somewhat higher immunity to fecal-oral transmission for two rather than one successful IPV dose, with any mix of successful IPV doses and live poliovirus infections resulting in the highest immunity state with respect to transmission potential [22]. Moreover, the DEB model assumes that IPV-induced immunity effectively limits oropharyngeal transmission [22], which we assume accounts for 30% of all transmissions in both modeled high-risk populations.

## Results

### Analysis I: comparison among serotypes of the vulnerability to OPV-related viruses after cessation

**Fig. 1:**
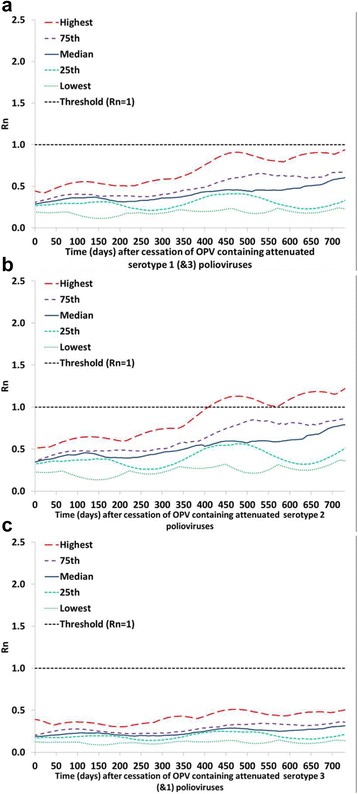
Comparison of the vulnerability to OPV virus after OPV cessation by serotype. Selected percentiles from the distribution of R_n_ values for OPV virus (stage 0) among 520 subpopulations that used OPV-only in 2013 in the global model [4] as a function of time after homotypic OPV cessation (analysis I). These base case results assume tOPV intensification before the tOPV-bOPV switch for serotype 2 (adopted from Duintjer Tebbens et al. 2016 [15]) and assume continued high maintenance of population immunity with bOPV SIAs until bOPV cessation for serotypes 1 and 3. **a** Serotype 1. **b** Serotype 2. **c** Serotype 3

**Fig. 2:**
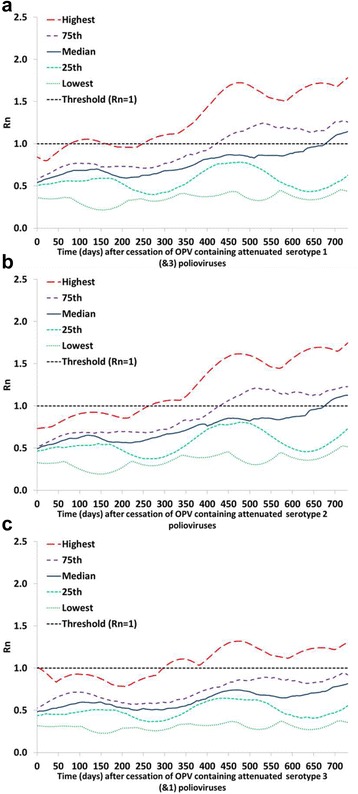
Comparison of the vulnerability to partially-reverted virus after OPV cessation by serotype. Selected percentiles from the distribution of R_n_ values for stage 10 partially-reverted OPV virus among 520 subpopulations that used OPV-only in 2013 in the global model [4] as a function of time after homotypic OPV cessation (analysis I). These base case results assume tOPV intensification before the tOPV-bOPV switch for serotype 2 (adopted from Duintjer Tebbens et al. 2016 [15]) and the assume continued high maintenance of population immunity with bOPV SIAs until bOPV cessation for serotypes 1 and 3. **a** Serotype 1. **b** Serotype 2. **c** Serotype 3

**Fig. 3:**
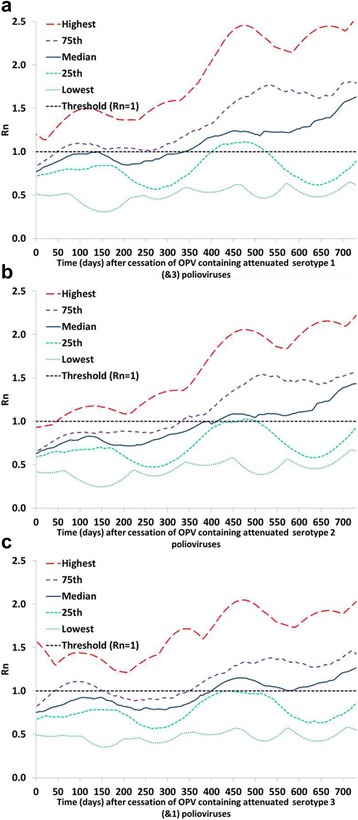
Comparison of the vulnerability to VDPV virus after OPV cessation by serotype. Selected percentiles from the distribution of R_n_ values for VDPV virus (stage 19) among 520 subpopulations that used OPV-only in 2013 in the global model [4] as a function of time after homotypic OPV cessation (analysis I). These base case results assume tOPV intensification before the tOPV-bOPV switch for serotype 2 (adopted from Duintjer Tebbens et al. 2016 [15]) and the assume continued high maintenance of population immunity with bOPV SIAs until bOPV cessation for serotypes 1 and 3. **a** Serotype 1. **b** Serotype 2. **c** Serotype 3

Figures 1, 2 and 3 (analysis I) show the serotype differences in the distribution of R_n_ values for reversion stages 0, 10, and 19, respectively, as a function of time after the tOPV-bOPV switch (for serotype 2) or bOPV cessation (for serotypes 1 and 3). The different curves in Figs. 1, 2 and 3 reflect selected percentiles of the distributions of R_n_ values among the 520 subpopulations in the global model that still used OPV in 2013 [4]. Thus, at each point in time after homotypic OPV cessation, each curve represents the one subpopulation that corresponds to the indicated percentile of the distribution of all 520 R_n_ values at that point in time (or the average of the two subpopulations closest to the percentile). Taken together, these percentiles illustrate the full range of when subpopulations become vulnerable to ongoing transmission of the specified polioviruses, with the highest curve showing the most vulnerable subpopulation and the lowest curve showing the least vulnerable subpopulation. We emphasize that these curves only illustrate the vulnerability as a function of time since OPV cessation. The risk in the event of a non-synchronous cessation depends on both the vulnerability and the exposure to OPV-related viruses from populations that continue to use OPV to populations that already stopped using OPV.

Looking at the OPV parent strains (Fig. 1), we see that among the three serotypes, subpopulations develop R_n_ values > 1 and thus become vulnerable to ongoing transmission of an OPV parent strain after OPV cessation the soonest for the serotype 2 OPV parent strain. This relates directly to the assumed high transmissibility of the serotype 2 OPV parent strain compared to the serotypes 1 and 3 OPV parent strains (Table 1). None of the subpopulations in the global model become vulnerable to ongoing transmission of the serotype 1 OPV parent strain within a two-year window, and vulnerability to ongoing transmission of the serotype 3 OPV parent strain remains even lower. Thus, Fig. 1 suggests a very long window until a failure to synchronize bOPV cessation would lead to a possibility that the OPV parent strains found in bOPV could continuously transmit in populations that stopped using bOPV. However, as mentioned in the methods section, subpopulations that use OPV typically generate some partially-reverted OPV-related viruses that can circulate independently of the OPV parent strains, even in the context of high population immunity and very limited secondary transmission of the OPV parent strains [15]. For example, for serotype 2 the model indicates that subpopulations with high immunity will typically experience ongoing transmission of OPV-related polioviruses up to reversion stage 8 and that subpopulations with low immunity will typically experience ongoing transmission of viruses well over stage 10, although these may not ultimately evolve to become fully-reverted cVDPVs [15]. For serotypes 1 and 3, which we assume do not evolve as quickly (Table 1), the model indicates that OPV-related viruses up to stage 5 or 6 may circulate in subpopulations with high enough population immunity to prevent the emergence of indigenous cVDPVs after bOPV cessation.

Figure 2 shows that subpopulations become vulnerable to ongoing transmission of partially-reverted OPV-related viruses in stage 10 somewhat sooner for serotype 1 than for serotypes 2 or 3. The higher vulnerability for serotype 1 reflects the lower level of population immunity to transmission typically sustained for serotype 1 compared to serotype 2 (because OPV results in more effective vaccinations and secondary immunity for serotype 2) and the higher R_0_ of serotype 1 OPV-related viruses in stage 10 compared to serotype 3 OPV-related viruses in stage 10. Higher vulnerability does not necessarily translate into higher risks of a non-synchronous cessation for serotype 1 than for serotypes 2 and 3 because non-synchronous serotype 2 cessation may involve a greater probability of exposure to higher reversion stages. However, Fig. 2 does not suggest a substantially lower risk associated with bOPV cessation than with the tOPV-bOPV switch.

Figure 3 shows when subpopulations become vulnerable to ongoing transmission of fully-reverted VDPVs (stage 19) and suggests the greatest initial vulnerability to serotype 3 VDPVs, but the greatest ultimate vulnerability to serotype 1 VDPVs. These findings reflect the generally low population immunity to transmission for serotype 3 compared to the other two serotypes [23], consistent with typical serological findings. Thus, if a serotype 1 or 3 cVDPV already exists in any subpopulation, then the risk of transmission following an importation of this virus into other subpopulations in the event of a non-synchronous bOPV cessation becomes very high very quickly.

### Analysis II: vulnerability to serotype 1 and 3 OPV-related viruses for different population immunity maintenance scenarios

**Fig. 4:**
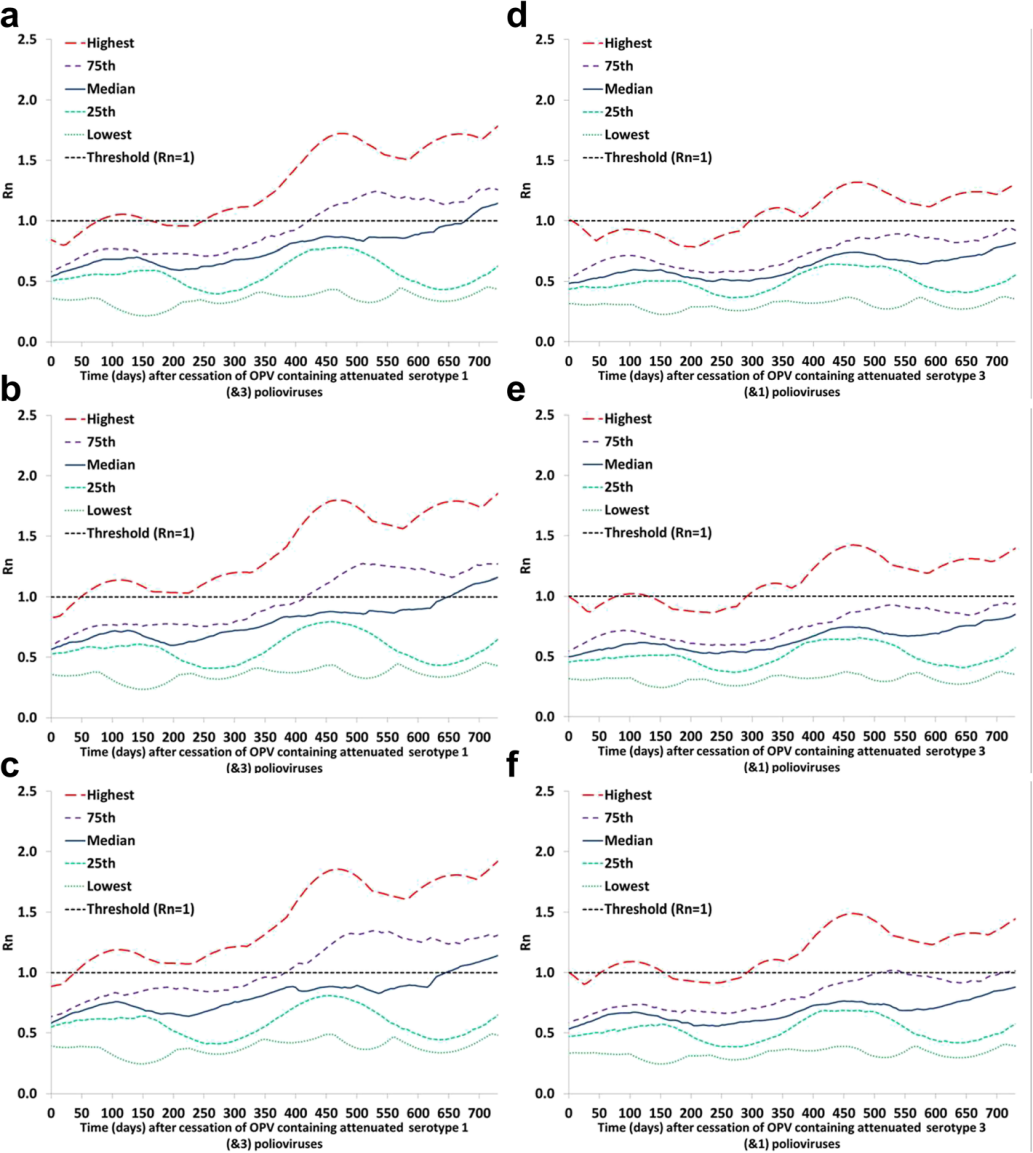
Vulnerability to partially-reverted virus after OPV cessation, by serotype and population immunity maintenance scenario. Selected percentiles from the distribution of R_n_ values for stage 10 OPV-related virus among 520 subpopulations that used OPV-only in 2013 in the global model [4] as a function of time after bOPV cessation for different scenarios of maintenance with bOPV SIAs until bOPV cessation (analysis II; scenarios adopted from Duintjer Tebbens and Thompson 2015 [29]). **a** Serotype 1, base case (high population immunity maintenance with bOPV). **b** Serotype 1, medium population immunity maintenance with bOPV. **c** Serotype 1, low population immunity maintenance with bOPV. **d** Serotype 3, base case (high population immunity maintenance with bOPV). **e** Serotype 3, medium population immunity maintenance with bOPV. **f** Serotype 3, low population immunity maintenance with bOPV

Figure 4 (analysis II) shows when subpopulations become vulnerable to ongoing transmission of partially-reverted OPV-related viruses in stage 10 under different scenarios for bOPV use until bOPV cessation (Table 2). These results suggest a relatively modest effect of pre-cessation bOPV use on vulnerability in the event of a non-synchronous bOPV cessation for the bOPV use scenarios considered. However, the low population immunity maintenance scenario involves the emergence of an indigenous serotype 1 cVDPV in one of the subpopulations after bOPV cessation. Although the resulting response subsequently lowers the R_n_ values for the “Highest” (red) curve in Fig. 4c, this scenario implies a programmatic failure. In contrast, no serotype 3 cVDPV occurs even with a large reduction in bOPV SIAs, and therefore none of the R_n_ curves include a decrease due to an outbreak. All decreases in R_n_ values for serotype 3 relate to seasonality (and consequent changes in which populations make up the different percentiles at different points in time) rather than outbreaks.

### Analysis III: comparison among serotypes of the risks of unauthorized post-cessation OPV use

**Fig. 5:**
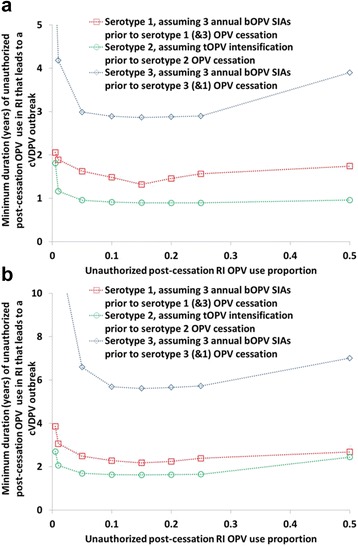
Comparison of the risks of unauthorized OPV use by serotype. Serotype comparison of the minimum duration after bOPV cessation or the tOPV-bOPV switch until unauthorized bOPV or tOPV use, respectively, leads to a cVDPV outbreak as a function of the extent of unauthorized use (analysis III). Unauthorized post-cessation RI OPV use proportion represents the proportion of covered children who receive a full schedule of the stopped OPV vaccine. **a** Population with properties like northern India. **b** Population with properties like northern Nigeria (y-axis range differ from panel **a**)

Figure 5 illustrates the results of analysis III on the potential impact of unauthorized post-cessation OPV use in RI for all three serotypes using comparable assumptions about pre-cessation SIAs and the extent of unauthorized use for the full RI schedule. The results suggest somewhat lower risks, i.e., longer times until unauthorized use would lead to a cVDPV outbreak, for serotype 1 than for serotype 2 and much lower risks for serotype 3. Assuming that three bOPV SIAs occur annually prior to bOPV cessation, unauthorized use of bOPV seems unlikely to cause cVDPV outbreaks during the first year after bOPV cessation even in an area as conducive to the spread of polioviruses as northern India (Fig. 5a). The relative differences among the three serotypes in risks from unauthorized OPV use appear similar across the modeled populations with properties like northern India and northern Nigeria (Fig. 5a and b). However, due to differences in those population properties, populations like the one in northern Nigeria would probably take twice as long as populations like the one in northern India to become vulnerable to unauthorized post-cessation OPV use potentially causing cVDPV outbreaks.

### Analysis IV: unauthorized OPV use for different assumptions about IPV and bOPV use

**Fig. 6:**
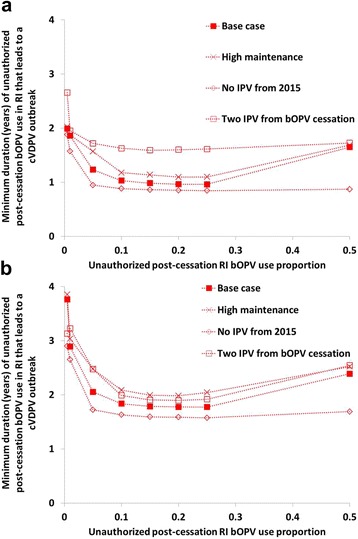
Serotype 1 risks of unauthorized bOPV use for different scenarios. Minimum duration after bOPV cessation until unauthorized bOPV use leads to a serotype 1 cVDPV outbreak as a function of the extent of unauthorized use for the base case and alternative scenarios (analysis IV). Unauthorized post-cessation RI bOPV use proportion represents the proportion of covered children who receive a single dose of bOPV instead of the scheduled IPV dose. **a** Population with properties like northern India. **b** Population with properties like northern Nigeria

Figure 6 suggests that for the base case (i.e., only one annual bOPV SIA between the tOPV-bOPV switch and OPV cessation, two bOPV SIAs in the year of bOPV cessation, and a single dose of IPV given in RI), populations with properties like northern India would experience a serotype 1 cVDPV outbreak if a relatively high level of unauthorized bOPV use, i.e., at least 5% of covered children, continues for approximately a year. However, unauthorized post-cessation bOPV use in northern India at very low levels, i.e., < 1% of covered children received bOPV, could continue for almost 2 years without causing serotype 1 cVDPV outbreaks. For a population with properties like northern Nigeria, the minimum duration of bOPV use that would result in a serotype 1 cVDPV outbreak equals approximately 1.5 years with a relatively high level of unauthorized bOPV use and three to 4 years at a very low level of unauthorized bOPV use. In addition, as shown in Fig. 6, a policy of high population immunity maintenance with bOPV SIAs, i.e., 3 annual SIAs only marginally decreases the risks compared to the analysis IV base case. A failure to use IPV from 2015 onward results in a small increase in risk of serotype 1 cVDPV outbreaks, although this increase in risk grows if the proportion of the children who receive bOPV reaches 50%. In the absence of any OPV use after bOPV cessation, adding a second IPV dose further reduces the risk of a serotype 1 cVDPV outbreak following unauthorized bOPV use. The risk from unauthorized bOPV use is reduced compared to the single IPV dose in the base case because the second dose provides more children with greater immunity to oropharyngeal transmission and, to a lesser extent, fecal-oral transmission, than a single IPV dose [22].

## Discussion

This study suggests generally similar risks associated with a non-synchronous global bOPV cessation or unauthorized bOPV use after bOPV cessation to those associated with the cessation of tOPV that occurred as part of the tOPV-bOPV switch. This finding is driven by the risks associated with ceasing use of OPV containing serotype 1 polioviruses because the risks associated with ceasing use of OPV containing serotype 3 polioviruses are lower than for OPV containing serotype 1 or 2 polioviruses. As with the tOPV-bOPV switch, post-cessation low levels of unauthorized use of bOPV could potentially continue for one or more years, depending on the area and degree of bOPV use, without causing new cVDPV outbreaks. However, this time becomes shorter in chronically under-vaccinated subpopulations within already high-risk settings [16]. Given the similarities in the risks involved between bOPV cessation and the cessation of tOPV use, bOPV cessation would benefit from risk reduction efforts similar to those used for the tOPV-bOPV switch.

The observation from this model that the risks from cVDPVs are similar for the cessation of OPV containing serotype 1 as for the cessation of OPV containing serotype 2 is consistent with patterns in VDPVs seen before 2005 when the GPEI began to use monovalent OPV and bOPV in many SIAs instead of tOPV. For example, serotype 1 and 2 cVDPVs resulted in similar numbers of identified outbreaks and cases of polio through 2005, with 4 outbreaks and 72 cases of polio caused by serotype 1 cVDPVs and 4 outbreaks and 40 cases of polio caused by serotype 2 cVDPVs [26, 31, 32]. While serotype 2 cVDPV outbreaks occurred with substantially greater frequency and severity than serotype 1 or 3 cVDPV outbreaks during 2006–2015 [31, 33], a pattern which helped motivate the tOPV-bOPV switch [2], this occurred primarily due to lower population immunity to serotype 2 transmission in many countries during that period. Moreover, in 2010 the Global Polio Laboratory Network changed to a less stringent definition of a VDPV for serotype 2 than the ones it uses for serotype 1 and 3 VDPVs [34]. For all poliovirus serotypes, low serotype-specific population immunity to transmission represents the main risk factor for cVDPVs [32]. The lower population immunity to serotype 2 transmission during 2006–2015 largely resulted from a shift in multiple countries to conducting SIAs with OPV that contained attenuated serotype 1 and/or 3 polioviruses but not attenuated serotype 2 polioviruses. This shift began in 2005 and continued through 2015, when the number of SIAs conducted with OPV containing attenuated serotype 2 polioviruses increased in preparation for the tOPV-bOPV switch [7]. Sub-optimal RI coverage with tOPV and prior elimination of WPV2, which was last detected in 1999, also contributed to inadequate population immunity to serotype 2 transmission [31, 33].

Prior modeling studies have shown that preventing the creation of indigenous cVDPVs after OPV cessation requires higher population immunity to transmission for serotype 2 than for serotype 1, but higher OPV coverage for serotype 1 than for serotype 2 [23]. This apparently counter-intuitive result relates primarily to the greater secondary spread of the attenuated serotype 2 polioviruses in OPV, and the resulting intestinal immunity against serotype 2 infections. Consistent with those findings, this work suggests similar overall risks from a lack of synchronization or unauthorized OPV use associated with the implementation of bOPV cessation as with the tOPV-bOPV switch. Thus, one should not assume that bOPV cessation comes with lower risks than the tOPV-bOPV switch. Synchronization of bOPV cessation within and across countries, minimizing unauthorized OPV use after cessation, and maintaining high population immunity to transmission until bOPV cessation are important strategies for minimizing the risks associated with bOPV cessation [21].

As long as use of all OPV containing attenuated serotypes 1 and 3 polioviruses ceases at the same time, i.e., as part of globally-coordinated bOPV cessation, the lower risks from ceasing use of OPV containing serotype 3 polioviruses will be overshadowed by the risks associated with ceasing use of OPV containing serotype 1 polioviruses. However, the characteristics of attenuated serotype 3 polioviruses become relevant if the global cessation of OPV containing attenuated serotype 3 polioviruses were separated from cessation of OPV containing serotype 1 polioviruses, perhaps in the form of a global switch from bOPV to monovalent OPV containing only attenuated serotype 1 polioviruses (mOPV1) [35, 36]. Based on the results of this study and current levels of population immunity for serotype 3, a bOPV-mOPV1 switch could occur with less tight synchronization, less stringent efforts to prevent unauthorized post-switch use of OPV containing attenuated type 3 polioviruses, and fewer pre-switch efforts to boost population immunity to type 3 poliovirus transmission than required for the tOPV-bOPV switch [7]. A bOPV-mOPV1 switch may become more attractive over time because the last reported serotype 3 WPV case occurred in 2012 [10], and continued OPV3 use leads to vaccine-associated paralytic poliomyelitis cases and may create new immunodeficiency-associated serotype 3 VDPV excretors [13, 37]. Nevertheless, any such bOPV-mOPV1 switch would ultimately still need to be followed by a globally coordinated cessation of mOPV1 use to address the problems posed by serotype 1 VDPVs, and all of the considerations that apply to bOPV cessation would apply to mOPV1 cessation.

Similar to the tOPV-bOPV switch, successful bOPV cessation will require global synchronization and efforts to verify the absence of bOPV from all supply chains. Gaps of several months or more in bOPV cessation dates come with an increased risk that OPV-related viruses will spread from countries or regions that still use bOPV to countries or regions that stopped using bOPV and then transmit and evolve to cVDPVs, leading to outbreaks. Early cessation of bOPV use appears particularly problematic in high-risk countries with environments conducive to the spread of polioviruses, such as countries with inadequate sanitation and tropical climates. The larger the populations that continue to use bOPV, and the longer they continue that use after the time that high-risk countries cease using bOPV, the greater the likelihood of exportations of OPV-related viruses to countries where OPV-related viruses can easily transmit and evolve to cVDPVs. Although few documented instances of the long-range spread of cVDPVs or of cVDPVs resulting from the long-range spread of OPV-related viruses have occurred to date [38], the rarity of such events does not imply zero risk of these possibilities in the future, just as the absence of cholera for at least 100 years in Haiti did not preclude a cholera outbreak following importation of cholera into Haiti from Nepal when the conditions favored its spread [39, 40]. The looser the synchronization of bOPV cessation, the more likely the occurrence of previously unobserved events, such as long-range spread of OPV-related viruses, particularly for serotype 1 OPV-related viruses. Moreover, if the GPEI deems it safe for some countries or regions that it supports to stop bOPV use earlier to save resources and stop vaccine-associated paralytic poliomyelitis cases, then this will raise questions why other countries or regions may not similarly discontinue bOPV to achieve these same benefits.

In addition to the epidemiological risks involved with a failure to globally synchronize bOPV cessation, a lack of synchronization would significantly complicate bOPV supply management [21, 23, 28] and could complicate efforts to secure cooperation from national governments in bOPV cessation [41, 42]. A lack of synchronization would cause countries that stop earlier to incur higher risks associated with importations of partially- or fully-reverted polioviruses from OPV compared to countries that stop later, mainly due to lower population immunity to transmission at the time of global cessation for the countries that stop earlier. We suspect that most countries, especially the ones at highest risk from polio outbreaks from cVDPVs, would conclude that the risks of stopping all OPV use prior to a coordinated global OPV cessation outweigh the benefits and would thus decline to do so.

With respect to unauthorized OPV use, environmental detections of viruses closely related to the attenuated serotype 2 polioviruses after the tOPV-bOPV switch and follow up investigations have suggested continued inadvertent use of tOPV in several countries [8, 21, 43]. These events further highlight the need for careful efforts, particularly by governments, to ensure complete bOPV withdrawal after its global cessation. IPV use could play a small role in decreasing risks from unauthorized bOPV use, with the cumulative effect on population immunity to serotype 1 transmission from IPV administered through RI over years being greater than the immediate effect of IPV on serotype 2 transmission at the time of the tOPV-bOPV switch [23, 30, 44, 45]. Although population immunity to serotype 1 transmission will inevitably decline after bOPV cessation, IPV use helps slow that decline, and two IPV doses per child will slow it more than a single dose. Whether this reduction in risk justifies the substantial investment to 2 IPV doses and whether sufficient IPV supply will exist remain open questions.

Although our models relied on an extensive calibration process to generate generic model inputs consistent with the evidence, the limitations of these models carry over to the analyses in this study [4, 17, 18, 22, 25, 26]. Global polio eradication remains a dynamic effort with frequent changes of plans. For example, the global model assumed tOPV intensification before the tOPV-bOPV switch and IPV introduction in all countries [4]. These things did not happen in all expected areas, and the failure to intensify tOPV use in some countries led to serotype 2 cVDPV outbreaks and necessary use of serotype 2 monovalent OPV for outbreak response [11, 20, 46–48]. Similarly, we remain uncertain about the future level of bOPV maintenance, the future IPV supply, and the timing of global serotype 1 WPV eradication, certification, and bOPV cessation. Thus, we expect the need to update the analyses when the plans for bOPV cessation become more definitive.

## Conclusions

This analysis demonstrates that the risks associated with bOPV cessation are similar to those associated with the tOPV-bOPV switch. Therefore, the global cessation of bOPV use should incorporate multiple risk reduction measures, including relatively tight synchronization of bOPV cessation within and across countries, minimizing unauthorized OPV use after cessation, and maintaining high population immunity to poliovirus transmission until bOPV cessation [21]. These measures would increase the likelihood of the world remaining truly polio free after the eradication of wild polioviruses.

## Abbreviations

bOPV: Bivalent OPV
cVDPV(2): (serotype 2) circulating VDPV
DEB: Differential equation-based
GPEI: Global Polio Eradication Initiative
IPV: Inactivated poliovirus vaccine
LPV: Live poliovirus
OPV: Oral poliovirus vaccine
R_0_: Basic reproduction number
RI: Routine immunization
R_n_: Mixing-adjusted net reproduction number
SIA: Supplemental immunization activity
tOPV: Trivalent OPV
VDPV: Vaccine-derived poliovirus
WPV: Wild poliovirus
